# Artificial Intelligence Tools in Dentistry: A Systematic Review on Their Application and Outcomes

**DOI:** 10.7759/cureus.85062

**Published:** 2025-05-29

**Authors:** Manjulika Tyagi, Shailesh Jain, Maitreyi Ranjan, Sahba Hassan, Nikhil Prakash, Dinesh Kumar, Ayush Kumar, Spriha Singh

**Affiliations:** 1 Prosthodontics, Subharti Dental College and Hospital, Swami Vivekanand Subharti University, Meerut, IND; 2 Prosthodontics, Bhojia Dental College and Hospital, Baddi, IND; 3 Microbiology, School of Dental Sciences, Sharda University, Greater Noida, IND; 4 Prosthodontics and Crown and Bridge, DJ College of Dental Sciences and Research, Modinagar, IND; 5 Prosthodontics, DJ College Of Dental Sciences and Research, Muradnagar, IND; 6 Oral Surgery, M.M. College of Dental Sciences and Research, Mullana, IND; 7 Dentistry, Dr. Harvansh Singh Judge Institute of Dental Sciences & Hospital, Panjab University, Chandigarh, IND; 8 Prosthodontics, KVG Dental College and Hospital, Sullia, IND

**Keywords:** artificial intelligence in dentistry, artificial neural networks, convolutional neural networks, deep learning, machine learning

## Abstract

Artificial intelligence (AI) in dentistry is rapidly getting recognized, altering the way that dentists treat patients and carry out clinical tasks. This systematic review investigates the integration of AI technologies into dental practice, with a particular focus on their impact on diagnostic accuracy, treatment planning, and operational efficiency. AI algorithms, especially those utilizing deep learning, have demonstrated exceptional proficiency in analyzing dental images, facilitating early and accurate detection of conditions such as caries, periodontal disease, and oral malignancies. Relevant literature was identified through an extensive search of electronic databases, including PubMed, Medline, Google Scholar, Scopus, and Web of Science, covering publications from 2019 to 2024. A total of 39 full-text articles were selected based on predefined inclusion and exclusion criteria, and their quality was assessed using the Quality Assessment of Diagnostic Accuracy Studies 2 (QUADAS-2) tool. From an initial pool of 342 articles, the 39 included studies revealed that AI-driven tools streamline administrative tasks, automate routine processes, and enhance overall practice efficiency, thereby reducing operational time. AI has exhibited significant potential in a wide range of dental fields, including radiology, periodontics, prosthodontics, orthodontics, oncology, diagnostic dentistry, and patient management. The findings highlight the promising role of AI in improving clinical outcomes and patient care while emphasizing the importance of balancing technological advancements with ethical considerations and human expertise. This review underscores the transformative potential of AI in modern dental practice and stresses the need for continued research and development in this dynamic and rapidly evolving field.

## Introduction and background

The application of artificial intelligence (AI) to dentistry represents a paradigm change in the industry and holds promise for improving many facets of dental practice and care. Dental practitioners' methods for diagnosing, treating, and managing oral health are wholly transformed by AI, which uses sophisticated algorithms and machine learning techniques to evaluate data and make judgments [1].

The term AI was coined by a mathematician, John McCarthy, in 1955 to characterize a machine's capacity to carry out intelligent activity [2]. AI is used today to describe any device or technology that can replicate human cognitive abilities, such as solving problems. Basic subcategories include machine learning, deep learning, cognitive computing, natural language processing, robotics, etc.

One common type of AI is called machine learning. This means a computer can learn to do something by looking at many examples. A more advanced version is called deep learning, where the computer uses many layers of "thinking steps" (called neural networks) to understand things such as pictures or patterns [3]. For example, deep learning can help a computer find signs of tooth decay in X-ray images. The primary purpose of these networks is to process complex images by extracting features from different layers of filters [4].

To detect abnormalities such as cavities, bone loss, or malignancies with high precision at an earlier stage, AI-powered tools are being utilized more efficiently than traditional approaches. AI can predict the likelihood of future oral diseases, allowing for early intervention and preventive care, based on historical data and patient-specific factors [5].

A clinical decision support system (CDSS) leverages a database of medical knowledge and algorithms to aid in decision-making and generate outcomes. This system enables healthcare professionals to work more efficiently, save time, and provide care at a reduced cost, thanks to its voice-activated controls and user-friendly interface.

AI is transforming the field of dentistry by enhancing diagnostic capabilities, personalizing treatment plans, predicting future dental issues, and improving operational efficiency. We anticipate that AI technology will continue to evolve, further revolutionizing dental practices and enhancing patient care [6].

## Review

Materials and methods

Data Sources

The Preferred Reporting Items for Systematic Reviews and Meta-Analyses (PRISMA) were consulted to conduct this systematic review and registered in the International Prospective Register of Systematic Reviews (PROSPERO) (ID CRD42024550755) [7]. The PICO (Problem/Patient/Population, Intervention, Comparison, and Outcome) question was framed to identify and structure the fundamental components of a systematic review.

Patient/population: Computer-aided design and computer-aided manufacture (CAD/CAM), radiographs (periapical, bitewing, orthopantomography, and cone-beam computed tomography), and two- and three-dimensional (3D) dental pictures.

Intervention: AI methods (deep learning, machine learning) are used in dentistry for treatment diagnosis, management, and prognosis.

Comparison: Dental experts, other digital analogs, and among AI models.

Outcome: Analysis of AI performance in terms of accuracy/precision, sensitivity, rating, and area under the curve (AUC) (Table 1).

**Table 1:** PICO framework PICO = Problem/Patient/Population, Intervention, Comparison, and Outcome

Criteria	Description
Problem/patient/population	Patient facial dental images (two-dimensional image (2D), three-dimensional (3D), radiographs(periapical, bitewing, orthopantomography, and cone-beam computed tomography), CAD/CAM (computer-aided design and computer-aided manufacturing)
Intervention	AI techniques (deep learning and machine learning) are applied in diagnosis, management, and predicting the prognosis of dental treatment
Comparison	Among AI models, dental experts and other digital methods
Outcome	Analysis of AI performance, accuracy/precision, sensitivity, rating, AUC: area under the curve, and AI applicability in different dental specialties

Study design type: For this review, we considered English-language published observational (case control and cohort), as well as interventional (trials) research.

Resource Selection

A thorough search was made in electronic databases such as PubMed/Medline, Google Scholar, Scopus, and Web of Science to qualify the literature for this paper. Terms such as dentistry, AI, deep learning, machine learning, artificial neural networks, and convolutional neural networks were used to assimilate the data from papers published over the past 10 years ( 2019-2024. The Mesh (Medical Subject Headings) terms and keywords were paired together with the use of Boolean operators "AND" and "OR" to construct the final search string (Table 2).

**Table 2:** Database search string

Database	Search String
PubMed/MEDLINE)	("Machine Learning" [MeSH] OR "Artificial Intelligence" [MeSH] OR "Deep Learning" [MeSH] OR "Computer Neural Networks" [MeSH] OR "Automated Pattern Recognition" [MeSH] OR "Natural Language Processing" [MeSH] OR "Artificial Neural Networks" OR “Machine Learning Algorithms” OR “Deep Neural Networks” OR “Computer-Aided Diagnosis” OR “AI” OR “ML” OR “DL”) AND (“Radiography, Dental" [MeSH] OR “Cone-Beam Computed Tomography" [MeSH] OR “Panoramic Radiography" [MeSH] OR “Dental Imaging” [MeSH] OR “Dental Radiology” [MeSH] OR “Dental Cone Beam Computed Tomography” OR “Dental Panoramic Radiography” OR “Dental Image Analysis” OR “Computer-Assisted Radiographic Image Interpretation” OR “3D Imaging”)
Scopus	TS=(“Artificial Intelligence” OR “Machine Learning” OR “Deep Learning” OR “Neural Networks” OR “Image Processing” OR “Pattern Recognition” OR “Natural Language Processing” OR “Artificial Neural Networks” OR “Machine Learning Algorithms” OR “Deep Neural Networks” OR “Computer-Aided Diagnosis” OR “AI” OR “ML” OR “DL”) AND TS=(“Radiography, Dental” OR “Cone-Beam Computed Tomography” OR “Panoramic Radiography” OR “Dental Radiography” OR “Dental Imaging” OR “Dental Radiology” OR “Dental Cone Beam Computed Tomography” OR “Dental Panoramic Radiography” OR “Dental Image Analysis” OR “Radiographic Image Interpretation, Computer-Assisted” OR ″3D Imaging” OR “Radiological Imaging”) AND TS=(“Dentistry” OR “Oral Medicine” OR “Oral Radiology” OR “Oral Diagnosis”)
Web of Science	TS=(“Artificial Intelligence” OR “Machine Learning” OR “Deep Learning” OR “Neural Networks” OR “Image Processing” OR “Pattern Recognition” OR “Natural Language Processing” OR “Artificial Neural Networks” OR “Machine Learning Algorithms” OR “Deep Neural Networks” OR “Computer-Aided Diagnosis” OR “AI” OR “ML” OR “DL”) AND TS=(“Radiography, Dental” OR “Cone-Beam Computed Tomography” OR “Panoramic Radiography” OR “Dental Radiography” OR “Dental Imaging” OR “Dental Radiology” OR “Dental Cone Beam Computed Tomography” OR “Dental Panoramic Radiography” OR “Dental Image Analysis” OR “Radiographic Image Interpretation, Computer-Assisted” OR ″3D Imaging” OR “Radiological Imaging”) AND TS=(“Dentistry” OR “Oral Medicine” OR “Oral Radiology” OR “Oral Diagnosis”)
Google Scholar	AI OR “Machine Learning” OR “Deep Learning” OR “Neural Networks” OR “Computer Vision” OR “Image Processing” OR “Pattern Recognition” OR “Natural Language Processing” OR “Artificial Neural Networks” OR “Machine Learning Algorithms” OR “Deep Neural Networks” OR “Computer-Aided Diagnosis” OR “ML” OR “DL” AND (“Radiography, Dental” OR “Cone-Beam Computed Tomography” OR “Panoramic Radiography” OR “Dental Radiography” OR “Dental Imaging” OR “Dental Radiology” OR “Dental Cone Beam Computed Tomography” OR “Dental Panoramic Radiography” OR “Dental Image Analysis” OR “Radiographic Image Interpretation, Computer-Assisted” OR ″3D Imaging” OR “Radiological Imaging”) AND (“Dentistry” OR “Oral Medicine” OR “Oral Radiology” OR “Oral Diagnosis”)

Full-length articles were retrieved. The journals were searched both manually and electronically. Two independent reviewers (MT and SJ) meticulously conducted the selection process to identify relevant studies. The selection process for the data required for this evaluation was divided into two stages. The articles were first chosen according to their titles and abstracts that were pertinent to the subject of our study. The preliminary search resulted in 342 articles that were appropriate enough to address the paper’s aim. Due to duplication, 192 articles were removed. Hence, we retrieved 150 articles for the second stage of selection for eligibility.

Eligibility Criteria

The following articles were examined to determine their eligibility for inclusion: (1) English-language papers, observational studies, clinical and nonclinical trials, and original research pertinent to AI in dentistry; (2) AI must be the primary focus of the article, and its application in dentistry should be addressed; and (3) there should be an appropriate description of the datasets utilized to evaluate a model.

Exclusion Criteria

The exclusion criteria are as follows: (1) articles unrelated to AI in dentistry; (2) unpublished articles; (3) articles with only abstracts; and (4) grey literature, case reports, letters to editors, review papers, and articles with fewer than ten participants or specimens were not included.

Study Risk of Bias and Quality Assessment

The quality and risk of bias of the included studies were assessed by the two independent reviewers. For this systematic review, the Quality Assessment of Diagnostic Accuracy Studies (QUADAS-2) tool was used to assess risk of bias and applicability of included studies [8]. Each reviewer evaluated the studies across predefined domains, and assessments were recorded separately. Discrepancies between reviewers were resolved through discussion. If consensus could not be reached, a third reviewer was consulted to make the final judgment. The following criteria were used to divide the risk of bias: total low risk of bias, if all the criteria were met; unclear risk of bias, if some criteria were met but at least one of them was only partially met; and high risk of bias, if at least one of the criteria was not fulfilled. "High risk of bias" would suggest "poor quality", and "low risk of bias" describes the "good-quality" grade.

Result

**Figure 1:**
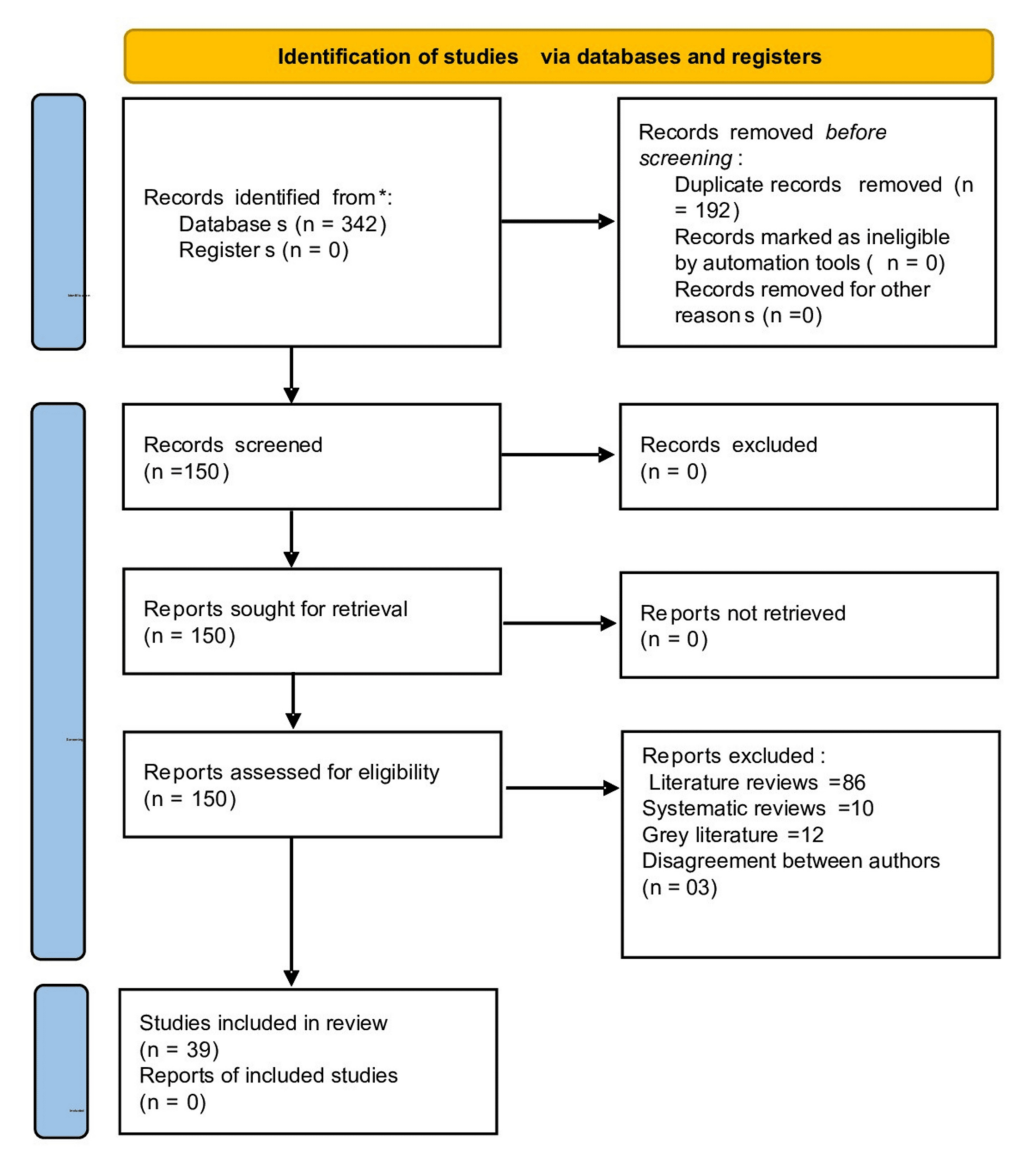
PRISMA flowchart PRISMA = Preferred Reporting Items for Systematic Reviews and Meta-Analyses

The primary search identified 342 articles based on key terms. Following those, 192 duplicates were removed, and 150 articles were screened based on titles and abstracts. The search was further narrowed down, and 111 irrelevant articles were excluded, which included literature reviews (86), systematic reviews (10), and grey literature (12). Additionally, three full-text articles were further excluded based on disagreement between authors. An eligibility assessment was conducted for the 39 full-text articles that remained. The 39 relevant articles were finally included and analyzed in the review. The literature search strategy is described through the PRISMA flowchart in Figure 1. Data has been extracted using a standardized data extraction form, which will include the following information: study characteristics (author, year, study design), comparison details(among AI algorithms and between algorithms and dental experts), and outcome measures in forms of effective, noneffective, neutral(measured in terms of efficiency of AI algorithms).

The general features of the included studies are summarized in Table 3 [9-47].

**Table 3:** Details of the studies that have used AI-based models are used in many dental specialties for diagnosis, treatment planning, clinical decision-making, and prognosis AUC = Area Under the Curve, ICC = Intraclass Correlation Coefficient, F = F-measure, VRF = Vertical Root Fracture, Positive/Negative Predictive Values = (PPV/NPV), BPNO = Binary Particle Swarm Optimization, ANNs = Artificial Neural Networks, CNNs = Convolutional Neural Networks, DCNNs = Deep Convolutional Neural Networks, BN = Bayesian Network, ROC = Receiver Operating Characteristic curve

Study authors/Year of publication	Algorithm architecture/Model	Objective of the study	Sample size	Diagnosis	Study factor	Evaluation	Comparator	Results	Treatment outcomes
Fati et al., 2022 [9]	CNN, SVM	Early diagnosis of OSCC by applying hybrid techniques based on fused features	5192	OSCC	Histopathological images	Accuracy: 99.1%, specificity: 99.6%, sensitivity: 99.5%, precision: 99.71%, AUC: 99.52%	Pathologist	(+)Effective	This method yielded promising results in histological image diagnostics for early diagnosis of OSCC.
Amin et al., 2021 [10]	DL: VGG16, InceptionV3, and Resnet	Improving the classiﬁcation of OSCC histopathological images into normal and cancerous classes by utilizing the concept of transfer learning through pre-trained CNN models	1224	OSCC	Histopathological images	The concatenated model achieved 96.66% accuracy (95.16% precision, 98.33% recall, and 95.00% specificity)	Single DL architecture	(+)Effective	The results demonstrate that the concatenated model can eﬀectively replace the use of a single DL architecture.
Casalegno et al., 2019 [11]	CNN	Detection and classification of dental lesion images obtained with near-infrared TI imaging.	217	Dental caries	Grayscale images	(IOU) score of 72.7% on a 5-class segmentation task	Experts	(+)Effective	This study demonstrated deep learning approach for the analysis of dental images holds promise for increasing the speed and accuracy of caries detection
Choi et al., 2021 [12]	CNN	To investigate the clinical utility of an AI diagnostic tool developed for TMJOA diagnosis from OPG	1189	TMJ disease	OPG	Accuracy: 0.78, sensitivity: 0.73, and specificity: 0.82	OMFS experts	(+)Effective	This study suggests that AI can play an important role in diagnosing TMJOA primarily from OPGs in most general practice clinics, where OMFR experts or CBCT are not available.
Geetha et al., 2020 [13]	BPNN	To develop an algorithm for a diagnostic system for the detection of dental care issues	105	Dental caries	IOPA	Accuracy: 97.1%, false-positive (FP) rate: 2.8%, receiver operating characteristic (ROC) area: 0.987, precision recall curve (PRC) area: 0.987	Dentist	(N)Neutral	This study suggests that dental caries can be predicted more accurately with a back-propagation neural network.
Mao et al., 2021 [14]	AlexNet, GoogleNet, Vgg19, and ResNet50	This study proposes a caries and lesion area analysis model based on CNN with transfer learning	1079	Dental caries	Bitewing radiograph	AlexNet model in this study for restoration and caries judgments has an accuracy as high as 95.56% and 90.30%, respectively	Experts	(+)Effective	This study can hopefully improve the accuracy of classification and further reduce the clinical time
Hiraiwa et al., 2019 [15]	Alexnet and Googlenet	The diagnostic performance of a deep learning system for classification of the root morphology of mandibular first molars on panoramic radiographs	760	Extra root	CBCT images	The AUCs of the deep learning systems using AlexNet and GoogleNet were 0.87 and 0.85, respectively, while that of the radiologists was 0.74	Radiologists	(+)Effective	High accuracy was demonstrated by the deep learning system in differentiating between one or more roots in the distal roots of mandibular first molars.
Zheng et al., 2021 [16]	(CNNs) (VGG19, Inception V3, and ResNet18)	To evaluate and compare three CNNs for analyzing the radiographic penetration depth of carious lesions	844	Deep caries	IOPA	ResNet18 demonstrated the best performance (accuracy = 0.82)	5 Dentists	(+)Effective	When combined with clinical parameters, ResNet18's multi-modal CNN showed noticeably improved performance and showed promise in diagnosing pulpitis and deep caries.
Yu et al., 2020 [17]	CNN	This study aims to provide a skeletal diagnostic system that is both reliable and accurate by integrating a convolutional neural network (CNN)	5890	Occlusion classification	Lateral cephalograms	With a clinical performance of 96.40%, the vertical classification demonstrated the highest accuracy	2 Orthodontists	(+)Effective	This study aimed to provide an automatic system that directly produces a diagnosis simply with X-ray inputs.
Vila-Blanco et al., 2020 [18]	CNN (DANet and DASNet)	In this work, two fully automatic methods to estimate the chronological age of a subject from the OPG image are proposed, which are less time-consuming	2289	Chronological age estimation	OPG	DASNet>DANet	Dental expert	(+)Effective	The DASNet method can be used to automatically and accurately predict someone’s chronological age, especially in young subjects with developing dentitions.
Ekert et al., 2019 [19]	CNN	To apply CNNs on panoramic radiographs to detect ALs while reducing the effort	2001	Detection of apical lesions	IOPA	The mean (SD) AUC of the CNN was 0.85 (0.04) at a sensitivity of 0.65 (0.12) and a specificity of 0.87 (0.04). The resulting PPV was 0.49 (0.10), and the NPV was 0.93 (0.03)	6 Experienced dentists	(N)Neutral	CNN demonstrated a satisfactory discriminatory ability to identify ALs on panoramic radiographs after being trained on a small amount of image data.
Ayidh Alqahtani et al., 2023 [20]	CNN	Validating an automated deep convolutional neural network (CNN)-based tool for the segmentation and classification of teeth with orthodontic brackets on CBCT images	215	Classification	CBCT scans	High accuracy: 100%, recall rate: 99.9%, precision: 99%, AUC: 0.99	Two experts	(+)Effective	The CNN-based tool was time-efficient and highly accurate in the presence of brackets for segmentation and classification
Verhelst et al., 2021 [21]	CNN	To develop and validate a layered deep learning algorithm that automatically creates three-dimensional (3D) surface models of the human mandible	160	Segmentation	CBCT scans	The IoU of the AI method was 94.6%	2 Experts	(+)Effective	This method produces 3D mandibular models more reliably and efficiently
Kim et al., 2022 [22]	CNN	(AI) model using cephalometric images for the classification of sagittal skeletal relationships	1574	Classification	Lateral cephalograms	Sensitivity: 0.94, specificity: 0.97, precision: 0.94, accuracy: 0.96	Not mentioned	(+)Effective	The performance of the current DCNN-based AI model was better than that of the automated-tracing AI software
Kim et al., 2023 [23]		To evaluate the performance of the automated skeletal maturation assessment system for Fishman’s skeletal maturity indicators (SMI) for use in the dental field	2593	Skeletal maturity indicator	Radiographs	Overall prediction accuracy was 0.599 with an MAE of 0.499	3 Orthodontists	(+)Effective	AI-based automated system for the assessment of Fishman’s SMI, which is widely used in the dental field, especially in orthodontics
Karhade et al., 2021 [24]	AutoML	To develop and evaluate an automated machine learning algorithm (AutoML) for children’s classification according to early childhood caries (ECC) status	6404	ECC	ECC	AUC: 0.80, Se: 0.73, PPV: 0.49	10 Clinical examiners	(N)Neutral	Automated machine learning early childhood caries classifiers can be valuable for ECC screening
You et al., 2020 [25]	CNN	To design a deep learning-based artificial intelligence (AI) model to detect plaque on primary teeth	886	Dental plaque	IOPA	MIoU (0.736 ± 0.174)	Pedodontist	(N)Neutral	An AI model can help in detecting dental plaque on primary teeth
Kim et al., 2019 [26]	CNN (DeNTNet)	For developing an automated diagnostic support system that detects periodontal bone loss in panoramic dental radiographs	12,179	Bone loss	OPG	F1 score of 0.75	Dental clinicians	(+)Effective	A fully automated method improves the efficiency of diagnosing PBL and reduces the workload.
Krois et al., 2019 [27]	CNN	To apply deep CNNs on dental radiographic imagery to detect PBL on image segments of panoramic dental radiographs	2001	Bone loss	OPG	(SD) classification accuracy of the CNN was 0.81 (0.02)	6 Dentists	(N)Neutral	A moderately complex CNN trained on a limited amount of labeled radiographic images showed at least similar diagnostic performance to experienced dentists in detecting PBL
Thanathornwong et al., 2020 [28]	Faster R-CNN	Using a deep learning-based object detection method to identify periodontally compromised teeth on digital panoramic radiographs	100	Bone loss	OPG	Model achieved sensitivity of 0.84, a specificity of 0.88, and an F-measure of 0.81	Periodontists	(+)Effective	A faster R-CNN may reduce diagnostic effort by saving assessment time and allowing automated screening documentation
Li et al. 2021 [29]	CNN	To employ deep learning (DL) algorithms for highly efficient and accurate disease detection by developing a multi-task learning model	3932	Dental diseases	Oral photographs	Area under the curve (AUC) for detecting: soft deposits = 78.57%, dental calculus = 80.11%, gingivitis = 87.11%	3 Board-certified dentists	(N)Neutral	Deep learning models hold promise for making it possible to use oral photos to screen for dental diseases in large populations at a reasonable cost
Martino et al., 2020 [30]	1. SegNet; 2. U-Net; 3. U-Net with VGG16 encoder; 4. U-Net with ResNet50 encoder	Performance analysis of four different deep learning-based pixel-wise methods	188	OSCC	Histopathological images	Not clear	Expert pathologists	(-)Non effective	A publicly available ORCA dataset was created, which will facilitate the development of new algorithms, boosting research
Deif et al., 2022 [31]	(DL) models (VGG16, AlexNet, ResNet50, and Inception V3)	Diagnosis of oral squamous cell carcinoma using deep neural networks	1224	OSCC	Histopathological images	An accuracy of 96.3% was obtained when using Inception V3	Not mentioned.	(+)Effective	This integrated AI model significantly contributes to improving the diagnostic efficiency of OCSCC patients while reducing the diagnostic costs
Kuwana et al., 2021 [32]	DL model: DetectNet	To determine the performance of a deep learning model in the detection of maxillary sinuses on panoramic radiographs	5872	Sinus lesions	OPG/CT/CBCT	Accuracies, sensitivities, and specificities for the diagnosis of maxillary sinusitis were 90–91%, 88–85%, and 91–96%, respectively. False-positive rates = 0.00.	Not mentioned	(+)Effective	The DL model effectively detects the maxillary lesions
Orhan et al., 2021 [33]	ML model	To propose a machine learning model and assess its ability to classify TMJ pathologies on MRI	214	TMJ pathologies	MRI images	The AUC, sensitivity, and specificity for the training set were 0.89 and 1, while those for the testing set were 0.77 and 0.74	2 Radiologists	(+)Effective	A machine learning model was proposed that can classify the condylar changes and TMJ disc displacements
Cantu et al., 2020 [34]	CNN	To apply deep learning to detect caries lesions	3686	Dental caries	Bitewing radiographs	Accuracy of 0.80, specificity of 0.83	4 Dental experts	(+)Effective	The trained CNN model performed significantly more accurately than the majority of dentists
Fukuda et al., 2019 [35]	CNN	To evaluate the use of the CNN system for detecting vertical root fracture (VRF)	300	Vertical root fracture	OPG	Recall was 0.75, precision 0.93, and F-measure 0.83	2 Radiologists and 1 endodontist	(+)Effective	An AI model for detecting vertical tooth fractures on panoramic radiography was developed
Patil et al., 2020 [36]	ANN	To evaluate the reliability of artificial neural networks as a gender prediction tool	509	Gender prediction	OPG	ANN exhibited a higher accuracy of 75%	Not mentioned	(+)Effective	Forensic sciences can use ANN, a good gender prediction tool, to get accurate results
Al-Sarem et al., 2022 [37]	CNN AlexNet, VGG16, VGG19, ResNet50, DenseNet169, and MobileNetV3	to enhance the 3D missing tooth area planning using pretrained deep learning models	500	Tooth detection	CBCT	Overall precision was above 0.90. DenseNet169 shows an F1-score of 0.94 and an accuracy of 93.3% among all models	Implant expert	(+)Effective	This model may represent a promising time-saving tool serving dental implantologists with a significant step toward automated dental implant planning
Kuwada et al., 2020 [38]	CNN AlexNet, VGG-16, and Detect-Net	To verify and compare the performance of AI models for classifying maxillary impacted supernumerary teeth (ISTs)	550	Supernumerary tooth	OPG	DetectNet showed that recall, precision, and F-measure were all 1.0	2 Radiologists	(+)Effective	AI models have the potential to provide diagnostic support in the interpretation of panoramic radiographs
Fontenele et al., 2023 [39]	CNN	To develop and assess the performance of for automated three-dimensional (3D) maxillary alveolar bone segmentation	141	Alveolar bone segmentation.	CBCT	Manual IoU > AI IoU = 95.0 > 92.0. Precision (%) 98.0 for manual > 93.0 for AI	2 Observers	(N)Neutral	The proposed AI-based tool can facilitate the digital workflow for oral implant planning by allowing accurate, time-efficient, and consistent segmentation of the alveolar bone
Shaheen et al., 2021 [40]	3D U-Net-based AI framework	To develop and validate a deep learning approach for automatic tooth segmentation and classification from CBCT images	186	Tooth segmentation and classification	CBCT	The AI model shows precision (0.98±0.02) and recall (0.83±0.05)	2 Experts	(N)Neutral	The proposed AI model might enable future diagnostic applications while reducing the workload
Kim et al., 2021 [41]	CNN models: ResNet-18, 34, 50, and 101	To investigate and report a method for improving the accuracy of predictive models according to the depth of the neural network	960		Lateral cephalograms	ResNet-18 showed higher prediction performance than other models: AUC: 97.9%, Accuracy: 93.8%, Sensitivity: 88.2%, Specificity: 96.6%	Not mentioned	(+)Effective	This paper provides suggestions on the characteristics of an AI model for prediction using medical images
Wood et al., 2023 [42]	Machine learning	The aim of this study was to create algorithms by using various ML techniques in order to predict the post-pubertal mandibular length and Y axis of growth in males and to compare their accuracies	163	Growth	Digital cephalometric radiographs	Accuracy ranges from 95.80% to 97.64% for mandibular growth and 96.60% to 98.34% for the Y-axis growth	1 Investigator	(N)Neutral	No significant difference was found among the accuracies of the ML techniques tested
Park et al., 2021 [43]	4 Prediction models by ML	To develop prediction models for ECC evaluated the performance was evaluated by comparing the ML-based models with a regression model	4195	ECC	DMFT	receiver operating characteristic (AUROC) between 0.774 and 0.785	Dentists	(+)Effective	This study proposed that prediction models could be the first step towards developing interventions that can be used to prevent ECC
Chang et al., 2020 [44]	DL	To develop an automatic method for staging periodontitis	340	Bone loss	OPG	Pearson correlation coefficient: 0.73, intraclass correlation value: 0.91	Oral radiologists	(+)Effective	The developed method can help dental professionals to diagnose and monitor periodontitis systematically and precisely on panoramic radiographs
Kurt Bayrakdar et al., 2020 [45]	CNN	To detect alveolar bone loss from dental panoramic radiography images by using an artificial intelligence (AI) system	2276	Bone loss	OPG	Sensitivity, specificity, precision, accuracy, and F1 score of 0.94, 0.88, 0.89, 0.91, and 0.91, respectively	Experienced oral radiologist and periodontist.	(+)Effective	CNN can be used to facilitate diagnosis and treatment planning by oral physicians in the future
Lee et al., 2020 [46]	CNN	To evaluate the efficacy of the deep CNN algorithm for the identification and classification of dental implant systems	5390 +5380	Implant system classification	OPG+IOPA	AUC = 0.971, 95% confidence interval 0.963–0.978)	Board-certified periodontist	(+)Effective	A deep CNN architecture was useful for the identification and classification of dental implant systems
Yamaguchi et al., 2019 [47]	CNN	To assess the usefulness of the convolutional neural network (CNN) method to predict the debonding probability of CAD/CAM composite crown	8640	Debonding	JPEG images	Prediction accuracy, precision, recall, and F-measure values were 98.5%, 97.0%, 100%, and 0.985, respectively. AUC was 0.998	Not mentioned	(+)Effective	The CNN method established in this study demonstrated considerably good performance in terms of predicting the debonding probability of a CAD/CAM CR

The included studies ranged from 2019 to 2024 and indicated a steady rise in AI-related dental research. In this review, a comparison has been shown between the traditional diagnosis methods and AI, as well as among the various AI algorithms used. These neural networks are used to process data from computer tomography (CT), cone beam computed tomography (CBCT), bitewing, lateral cephalometric, facial, and panoramic radiographs (OPG) images. They also assist in identifying patterns or relationships within a given dataset.

Quality Assessment

**Figure 2:**
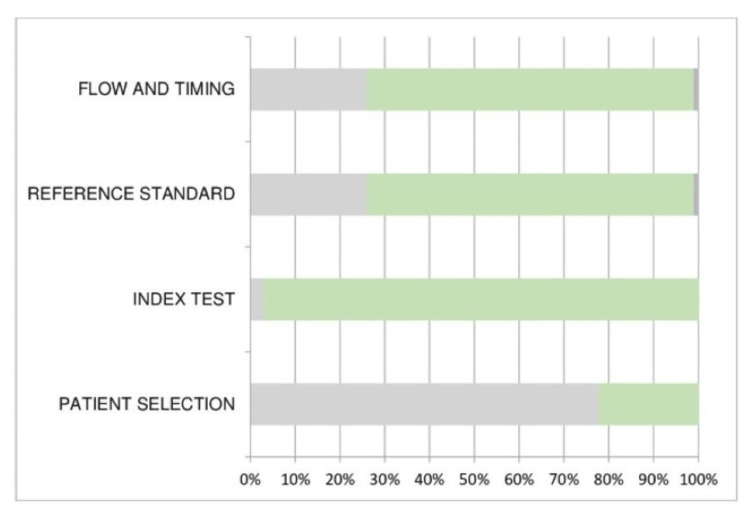
Risk of bias

**Figure 3:**
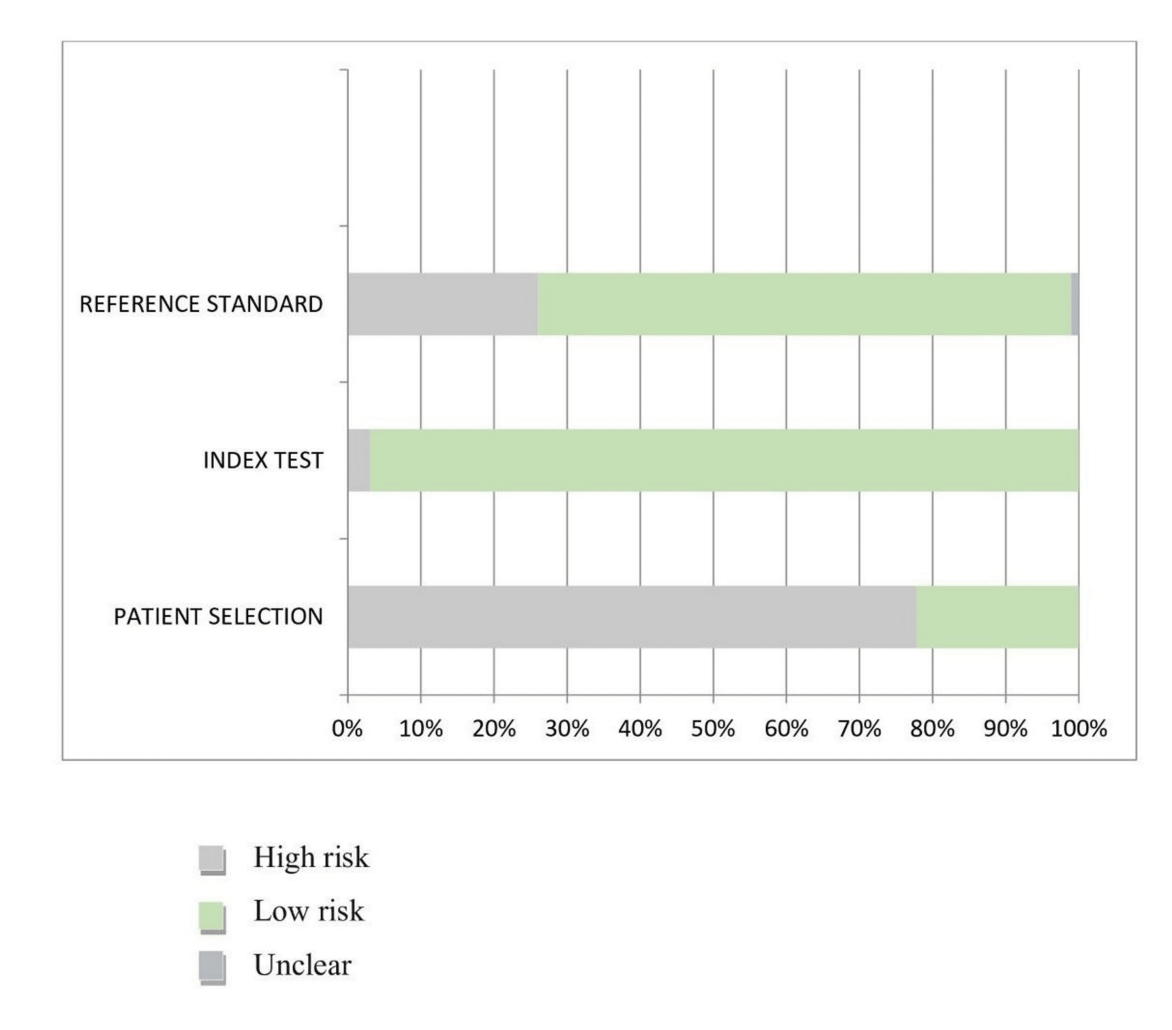
Applicability concerns

The modified QUADAS-2 tool was used to evaluate the methodological quality and risk of bias of the included studies. A high risk of bias for patient selection was reported by 77.20% of the studies. The reason could be the collection of data from the same center in most studies. The inclusion and exclusion criteria were also not explicitly explained in most studies. Since the data in most studies have undergone rigorous validation and standardized protocols were implemented, the reference standard has a low risk of bias. The index test (97.43%) and timings (76.92%) in the current systematic review showed a low risk of bias. Under the applicability domain, the risk is low-biased since the dataset is well-defined with the intended use of the index test. Additionally, the review question was explained by the index test's administration and interpretation (Figure 2 and Figure 3).

Discussion

AI has the potential to transform dentistry by improving diagnostics, treatment planning, patient management, and more. As technology advances, AI will likely become an even more integral part of dental care. Research indicates that the application of AI in dentistry benefits dentists throughout key stages of patient care, including diagnosis, treatment planning, and follow-up management. For instance, using a neural network to check for precancerous conditions and oral cancer is advantageous. They can help detect dental issues such as tumors that might be missed by the human eye or histopathological images. Suliman et al. [9] stated that an ANN-based hybrid model, ResNeT, provides an accuracy of 99.3% for detecting OSSC. In another study, deep learning models were used, and it was concluded that the DL model Inception V3 used with BPSO and the concatenated models using VGG16, InceptionV3, and Resnet provide better results with the best classification accuracy. In another study, a CNN model was used to increase the speed and accuracy of caries detection on a grayscale image. In a different diagnostic investigation, deep learning models, DetectNet and ResNet, were used to detect diseases of the maxillary region and TMJ osteoarthritis. In a study by Geetha et al. [13], five diagnostic algorithm systems were evaluated for diagnosing the depth of dental caries, among which, the back propagation method proved to be more accurate in classifying and detecting the caries. Four DL models are compared using AI technology to diagnose dental caries, with AlexNet offering the highest accuracy for caries detection. In a study, the ML-based model accurately predicted the post-pubertal maxillary and mandibular lengths. Another CNN model, ResNet18, showed enhanced performance in diagnosing deep caries and pulpitis. In another study, a CNN model provides good results for skeletal orthodontic diagnosis. Among the two CNN models, DANet and DASNet, for determining chronological age, DASNet outperforms DANet. A CNN model for apical lesion detection showed satisfying results. The CNN-based models also outperform the other state-of-the-art algorithms used for the classification and segmentation of teeth with brackets and maxillary alveolar bone. In one of the studies, the 3D U-Net architecture algorithm allowed the classification and segmentation of the images accurately and efficiently. A deep convolutional neural network (DCNN)-based AI model, when compared to automated tracing AI software, proved better with 0.96 accuracy. In another study, a hybrid skeletal maturity indicator, when enhanced by AI, gives clinically reliable results with 0.772 accuracy. In a study by Kharade et al. [24], an automated machine learning algorithm, Auto ML, gives the highest AUC for ECC detection. In a study for plaque detection in children, a CNN model was adapted, which showed clinically acceptable performance. In several other studies, the models of CNN DeNT Net, Deep CNN, hybrid models, Google Inception V3, and faster regional CNN (faster-RCNN ) performed satisfactorily in detecting periodontally compromised teeth. In one of the studies, a CNN model enables the screening of dental diseases among a large population. In another study, Martino et al. [30] contributed by creating the Oral Cancer Annotated (ORCA) dataset using the AI models, which can be used by other researchers for testing their approaches in the future. Another study on AI models demonstrates that the integration of AI models significantly contributes to improving the diagnostic efficiency of OCSCC patients while reducing the diagnostic costs. Kuwana et al. [32] in a study determined the performance of a deep learning model in the detection of maxillary sinuses on panoramic radiographs, which worked effectively.

Orhan et al. [33], in their study, used machine learning algorithms KNN and RF for better classifying condylar changes and TMJ displacements. Another study by Cantu et al. [32] proved that a CNN model, U-Net, proved to be more accurate than a dentist for caries detection. In a study done to determine vertical root fracture, the CNN system DetectNet provides a precision of 0.98. In forensic dentistry, an ANN model proved to be better for gender prediction with an accuracy of 75%. The CNN model DenseNet169 shows a superior precision of 0.98 for detecting missing tooth position and proved to be a time-saving tool among the six models used. Among the 3 DL models used for classifying maxillary impacted supernumerary teeth, DetectNet produced the highest values of diagnostic efficacy. Fontenele et al. [39], in their study, developed and assessed the performance of an automated three-dimensional (3D) maxillary alveolar bone segmentation AI-based tool that can facilitate the digital workflow for oral implant planning by enabling precise, reliable, and time-efficient alveolar bone segmentation. Shaheen et al. developed and validated a deep learning approach for automatic tooth segmentation and classification from CBCT images that might enable future diagnostic applications while reducing the workload. A CNN model, ResNet-18, outperforms other algorithms for the diagnosis of orthognathic surgery in one of the studies. Wood et al. in their study create algorithms by using various ML techniques to predict the post-pubertal mandibular length and conclude that no significant difference was found among the accuracies of the ML techniques tested. In one of the studies, the comparison with the traditional regression model machine learning-based prediction models showed favorable performance and can be used to predict early childhood caries, identify ECC high-risk groups, and implement active preventive treatments. Chang et al. [44] developed an automatic method for staging periodontitis that can help dental professionals diagnose and monitor periodontitis systematically and precisely on panoramic radiographs. Kurt Bayrakdar et al. [45] detected alveolar bone loss from dental panoramic radiography images using an AI system and proposed that CNN can be used to facilitate diagnosis and treatment planning by oral physicians in the future. In terms of classification accuracies, a CNN-based model showed comparable results for the classification of dental implant systems. The prediction of the debonding probability of CAD/CAM crowns from 3D models was successfully made by the deep learning CNN model, giving 98.5% accuracy.

## Conclusions

The integration of AI into dentistry marks a transformative advancement, offering substantial improvements in diagnostic accuracy, treatment planning, and overall patient care. Despite its benefits, the incorporation of AI also presents challenges, such as the need for stringent data privacy measures, the risk of over-reliance on technology, and the importance of continuous validation to ensure AI systems maintain their accuracy and effectiveness over time. In conclusion, while AI cannot replace the human element in dentistry, it acts as a powerful adjunct, enhancing clinical practice through more precise diagnoses, personalized treatment plans, and improved patient outcomes. Striking a balance between the application of AI and human expertise, while addressing ethical considerations, will be essential as this technology continues to evolve and become further integrated into dental care.
